# About the Genetic Contribution to Chronic Dizziness and Episodic Vertigo

**DOI:** 10.1007/s10162-023-00921-2

**Published:** 2023-12-20

**Authors:** Jose A. Lopez-Escamez

**Affiliations:** 1 Meniere’s Disease Neuroscience Research Program, Faculty of Medicine and Health, School of Medical Sciences, The Kolling Institute, University of Sydney, 2065 Sydney, New South Wales, Australia; 2 Otology and Neurotology Group CTS495, Instituto de Investigación Biosanitaria, Ibs.GRANADA, Universidad de Granada, 18071 Granada, Spain; 3 Division of Otolaryngology, Department of Surgery, Universidad de Granada, 18016 Granada, Spain; 4 Sensorineural Pathology Programme, Centro de Investigación Biomédica en Red en Enfermedades Raras, CIBERER, 28029 Madrid, Spain

**Keywords:** Genetic diseases, Vertigo, Dizziness, Common variants, Rare variants

When investigating the genetic underpinnings of any particular trait, there are two prime aspects to consider. Firstly, the condition has to demonstrate some degree of heritability, which is the proportion of phenotypic variance explained by genetic factors [1]. This can be reasonably addressed using genetic epidemiology, including familial aggregation, adoptee, and twin studies [2]. Secondly, the prevalence of the target phenotype is essential to anticipate the genetic architecture of the condition and identify causal genomic variation. Common disorders, with a prevalence > 5%, have a multiallelic (polygenic) structure with many common variants driving the association, each of them with a small effect size. Conversely, rare disorders are most often associated with few rare variants of large effect size [3].

The genetics of balance disorders is a challenging research area, complexified by the numerous anatomical structures involved in the balance system, including peripheral and central components, and the subsequently high heterogeneity of phenotypes. Peripheral origins of vertigo include benign paroxysmal positional vertigo (BPPV), Menière’s disease, and vestibular neuritis whereas central causes include motion sickness, vestibular migraine, and brainstem and cerebellar ischemia. This challenge in the definitions of vestibular disorders is evidenced with the rather low and variable heritability of balance impairment, ranging from 27 to 47% according to twin studies [4, 5]. Planning a genetic study in the older adult population faces some challenges. Epigenetic and environmental factors, along the life, add interindividual heterogeneity in the elderly cohorts. The aging population suffers from multiple comorbidities, they are usually receiving multiple drugs, and it is difficult to control all confounders associated with brain aging (i.e., cognition, behavior). Recently, Skuladottir et al. reported the first genome-wide association study (GWAS) on vertigo with 48,072 cases and 894,541 controls [6], providing an initial basis for the common genetic variations contributing to vertigo.

In this issue of *JARO*, Clifford et al. (2024) address some knowledge gaps in the field and contribute with a large GWAS on chronic dizziness in the elderly using 50,339 cases and 366,900 controls from the Million Veteran Research Program [7]. Chronic dizziness is a common complaint in the elderly and a broader term than vertigo. According to this definition, the “umbrella chronic dizziness” cohort would include elderly patients with visual, cerebellar, and proprioceptive deficits and cardiovascular (hypertension, diabetes), neurological (neurodegenerative disorder), and psychiatric disorders (depression, other mood disorders). The overlap of “chronic dizziness” with several comorbidities frequently found in the elderly population makes the interpretation of non-coding variants in GWAS difficult. In this study, Clifford et al. thus performed a drastic exclusion of episodic and recurrent imbalance traits such as BPPV and other vestibular disorders such as traumatic brain injury, Menière’s disease, or vestibular neuritis. They also excluded ataxias, i.e., Parkinson’s disease and cerebrovascular events. SNP heritability (*h*_s_^SNP^) was estimated at 0.059, indicating that the identified SNVs explained nearly 6% of the trait. They identified several intronic single nucleotide variants in the genes *MLLT10*, *BPTF*, and *LINCO1224*, but the functional role of these variants remains unknown.

Due to the lack of additional cohorts to be used for replication purposes, the authors compared their findings with the GWAS of Skuladottir et al. (2021), who reported an association with six common missense variants in *ZNF91*, *OTOGL*, *OTOG*, and *TECTA* and a cis-eQTL for *ARMC9*, the two first ones being driven by BPPV [6]. Some of these genes, such as *OTOG* and *TECTA* have also been identified in familial Menière’s disease by exome sequencing and segregation analyses [8]. However, none of these “cochlea-vestibular genes” were found in Clifford et al.’s study. Despite these top polymorphisms diverging in the two studies, the genetic correlation between the “chronic dizziness” study from Clifford et al. and the “vertigo” study from Skuladottir et al. was strong (rg = 0.67, *p*-value = 5.34 × 10^−20^), indicating shared common genetic mechanisms between the chronic and more acute vestibular symptoms.

Phenotyping precision is a critical issue, and studies based on self-reported symptoms usually have low-quality data, which is a limitation in complex disorders when retrospective data are retrieved from biobanks. This “minimum phenotyping” may explain the lack of replication of many GWAS in complex disorders [9]. Here, the two studies from Skuladottir et al. and those from Clifford et al. have addressed this challenge by using International Classification of Diseases’ coded diagnosis. Despite the different phenotypes being assessed, the two GWAS have a rather similar design and robust statistical support. The divergent results illustrate that precise phenotyping is crucial to reduce the granularity in available and prospective genetic data. Sample size does matter, but the diagnostic criteria and the precise phenotyping are critical factors for obtaining a reliable association, something which is challenging in the vestibular field when exploring the very limited number of existing GWAS datasets. The Barany Society International Classification of Vestibular Disorders has developed consensus documents with diagnostic criteria for the most vestibular disorders (https://www.thebaranysociety.org/icvd-consensus-documents), including not only episodic syndromes (i.e., vestibular migraine [10], Menière’s disease [11], motion sickness [12]) but also chronic syndromes such as persistent postural-perceptual disorder [13], mal de debarquement [14], and bilateral vestibulopathy [15]. These would deserve full consideration across existing and future biobanks to improve the precision, the performance, and reliability of future GWAS in the field and thus help disentangling the genetics between chronic and acute vestibular disorders. Finally, expression datasets from animal and human vestibular tissue, still unavailable to the scientific community, may help providing mechanistic insights into the origins of these disorders.
